# Risk for Suicidal Behavior After Psychiatric Hospitalization Among Sexual and Gender Minority Patients

**DOI:** 10.1001/jamanetworkopen.2023.33060

**Published:** 2023-09-08

**Authors:** Brian C. Thoma, Emily Hone, Alyssa Roig, Elijah Goodfriend, EJ Jardas, Bradley Brummitt, Sarah Riston, Dara Sakolsky, Jamie Zelazny, Anna L. Marsland, Kehui Chen, Antoine B. Douaihy, David A. Brent, Nadine M. Melhem

**Affiliations:** 1 Department of Psychiatry, University of Pittsburgh School of Medicine, Pittsburgh, Pennsylvania; 2 University of Pittsburgh Medical Center, Pittsburgh, Pennsylvania; 3 Department of Psychology, University of Pittsburgh, Pittsburgh, Pennsylvania; 4 Department of Statistics, University of Pittsburgh, Pittsburgh, Pennsylvania

## Abstract

**Importance:**

The months following inpatient psychiatric hospitalization are a period of high risk for suicidal behavior. Sexual and gender minority (SGM) individuals have elevated risk for suicidal behavior, but no prior research has examined whether SGM inpatients have disproportionate risk for suicidal behavior following discharge from psychiatric hospitalization.

**Objectives:**

To evaluate whether SGM patients have elevated risk for suicidal behavior following discharge from psychiatric hospitalization compared with heterosexual and cisgender patients and to examine whether differences in risk across groups were accounted for by demographic characteristics and clinical factors known to be associated with suicidal behavior.

**Design, Setting, and Participants:**

This prospective cohort study was conducted from August 2017 to July 2021 among inpatients aged 18 to 30 years who were voluntarily enrolled during psychiatric hospitalization. The study was conducted at an inpatient psychiatric hospital, with prospective data collected via follow-up visits and electronic health records.

**Main Outcomes and Measures:**

Onset and/or recurrence of suicidal behavior following discharge from psychiatric hospitalization, assessed at follow-up visits and through electronic health records.

**Results:**

A total of 160 patients were included, with 56 sexual minority (SM) and 15 gender minority (GM) patients. The median (IQR) age of the patients was 23.5 (20.4-27.6) years, 77 (48%) reported male sex assigned at birth, and 114 (71%) identified their race as White. During the follow-up period, 33 suicidal behavior events occurred (among 21% of patients). SM (hazard ratio [HR], 2.02; 95% CI, CI, 1.02-4.00; log-rank *P* = .04) and GM (HR, 4.27; 95% CI, 1.75-10.40; log-rank *P* < .001) patients had significantly higher risk for suicidal behavior compared with their heterosexual and cisgender counterparts, respectively, in bivariable analyses. Risk between SM and heterosexual patients was not different after controlling for demographic characteristics and clinical factors associated with suicidal behavior. GM patients exhibited elevated risk during the 100 days following discharge even after controlling for demographic and clinical characteristics (HR, 3.80; 95% CI, 1.18-11.19; *P* = .03).

**Conclusions and Relevance:**

Within this cohort study of psychiatric patients, SGM patients had higher risk for suicidal behavior than non-SGM patients following discharge. While SM patients’ risk was accounted for by clinical characteristics, GM patients’ risk for suicidal behavior was not accounted for by their acute psychiatric state on admission. Future studies with larger subsamples of GM individuals are needed, and inpatient clinicians must attend to the unique needs of SGM individuals to ensure they receive affirming services.

## Key Points

**Question:**

Do sexual and gender minority (SGM) patients have elevated risk for suicidal behavior following psychiatric hospitalization compared with heterosexual and cisgender individuals?

**Findings:**

In this cohort study of 160 young adults, risk for suicidal behavior was higher among SGM patients, and demographic and clinical characteristics did not account for differences between gender minority and cisgender patients in the 100 days following discharge.

**Meaning:**

Given the disproportionate representation of SGM patients in inpatient psychiatric populations as well as their increased risk for suicidal behavior following discharge from psychiatric care, additional attention to validating and effective care for SGM patients within inpatient psychiatric settings is warranted.

## Teaser

This cohort study evaluates whether sexual and gender minority (SGM) patients have elevated risk for suicidal behavior following discharge from psychiatric hospitalization compared with heterosexual and cisgender patients.

## Introduction

Suicide is a leading cause of death in the United States,^1^ and the year following inpatient psychiatric hospitalization is characterized by markedly increased risk for suicidal behavior (SB) among psychiatric patients.^2,3,4,5,6^ The suicide rate is highest within 3 months immediately following hospital discharge among patients with suicidal ideation or SB on admission to psychiatric care.^2^ SB is defined as engaging in behaviors to intentionally end one’s own life, and prior history of SB, and mood, anxiety, substance use, and posttraumatic stress disorders contribute to risk for future SB within this population.^1,7,8,9^ Psychosocial stressors also contribute to risk for SB among psychiatric patients, including history of childhood abuse and acute stress.^1,4^ While elevated risk for SB following discharge is well-documented, little is known about the risk among sexual and gender minority (SGM) individuals during this critical time period, a population at disproportionately high-risk for SB across the lifespan.

Sexual minority (SM) individuals (who identify as lesbian, gay, bisexual, or queer) have higher risk for SB compared with heterosexual individuals.^10,11,12^ Similarly, gender minority (GM) individuals (whose gender identity is different than their sex assigned at birth) have higher risk for SB compared with cisgender individuals (whose gender identity is the same as their sex assigned at birth).^13,14,15^ While SGM individuals experience heightened risk for SB across the lifespan, their risk is particularly high during adolescence and young adulthood.^13,16^ SGM individuals experience minority stress related to sexual orientation and/or gender identity, including prejudice events, stress related to concealment and disclosure of identity, shame about their stigmatized identities, and expectations of rejection from others.^17,18,19^ Minority stress is also associated with higher rates of psychopathology within SGM populations,^12,18,20,21^ and elevated rates of psychopathology contribute to their increased risk for SB.^22^

There is a paucity of research on risk for SB among SGM individuals following psychiatric hospitalization. Only one prior study has examined such risk following visits to emergency department (ED) services^23^ and found 25% of SGM patients aged 13 to 25 years reported SB in the year following their ED visit, a risk that did not differ from non-SGM individuals. No prior research has examined risk for SB among SGM individuals following discharge from psychiatric inpatient care, and no prior work has examined risk among SM and GM individuals separately.

In the current study, our primary objective was to examine risk of SB among SM and GM individuals compared with heterosexual and cisgender individuals, respectively, following discharge from psychiatric hospitalization in a sample of psychiatric inpatients aged 18 to 30 years. We also examined whether risk among SGM individuals is higher after controlling for prior history of SB, severity of suicidal ideation, current psychiatric symptoms, and psychosocial stressors at the time of admission, which are known to be factors associated with SB and could account for higher risk for SB among SGM individuals following discharge. We hypothesized that SM and GM individuals would show higher risk for SB following discharge compared with heterosexual and cisgender individuals, respectively, even after controlling for other factors associated with SB.

## Methods

### Sample

Participants included psychiatric inpatients aged 18 to 30 years admitted to Western Psychiatric Hospital (Pittsburgh, Pennsylvania). Patients were recruited as part of a larger study investigating biological predictors in the hypothalamic-pituitary-adrenal axis and inflammatory pathways for SB in young adults. Psychiatric inpatients across the spectrum of psychopathology and SB were recruited. Of 207 psychiatric inpatients participating in the larger study at the time of these analyses, 160 provided information on sexual orientation and gender identity and were included in the current analysis: 56 patients identified as SM (35%) and 15 (9%) identified as GM. Most GM patients also identified as SM (14 [93.3%]). Participants who were included in the present analysis were more likely to have anxiety and posttraumatic stress disorder (PTSD) diagnoses compared with those excluded due to lack of sexual orientation and gender identity information.

Given the focus on biological markers in the larger study, patients were excluded if they had chronic inflammatory diseases, were taking corticosteroids or other medications that affect inflammatory or neuroendocrine markers, or if they were pregnant. Patients with limited cognitive ability to provide consent were excluded. Attending physicians were contacted to confirm eligibility. Once approved, members of the treatment team approached the patient to assess their interest in the study. Participation was voluntary, and all participants provided written informed consent. Participants were paid $95 for completing all baseline procedures, including $35 for completing the clinical interview. The study received approval from the University of Pittsburgh institutional review board.

### Assessments

#### Demographic Characteristics

Sexual orientation was assessed with 1 item querying self-identity with the following response options: “Heterosexual or straight,” “Gay or lesbian,” “Bisexual,” “Other,” and “Not sure.” Patients were coded SM if they endorsed any sexual orientation other than “Heterosexual or straight,” including “Not sure.” Individuals who endorsed “Not sure” were included in the SM group because individuals who are questioning their sexual orientation have similar, or even higher, risk for SB when compared with other SM individuals, and questioning individuals are typically included in the SM group in health equity research.^24^ Gender identity was assessed with 2 items, one assessing biological sex at birth (response options included “Female,” “Male,” and “Other”) and one assessing gender identity (response options included “Female,” “Male,” “Transgender,” “Other,” and “Not sure.”) Patients were coded GM if they endorsed a gender identity that was different than their sex assigned at birth or if they identified as “Transgender” or with an “Other” gender identity. Age and race were also assessed using self-report survey measures, consistent with measures required by the funding agency.

#### Psychiatric Disorders

We used the Structured Clinical Interview for *DSM-5* disorders (SCID-5)^25^ to assess current and lifetime history of psychiatric disorders. Masters-level clinical interviewers conducted interviews after receiving training to administer the SCID-5. Consensus diagnoses were conducted with a psychiatrist (D.S.) using all sources of information. We used the Longitudinal Interval Follow-Up Evaluation (LIFE)^26^ to record variations of symptoms over weekly or monthly intervals (depending on follow-up period) to assess change in symptoms. We also collected data from electronic health records (EHRs) after receiving Health Insurance Portability and Accountability Act authorization during the consent process. Psychiatric disorders were determined based on SCID-5 diagnoses and/or EHR data, and all diagnoses were added to data set, even if they were recorded only during study visits or in EHR data.

#### Suicidal Ideation and Behavior

Lifetime and current suicidal ideation and SB were assessed at baseline using the Columbia Suicide Severity Rating Scale (C-SSRS).^27^ The C-SSRS evidences excellent internal consistency within samples of psychiatric inpatients (α = .95).^28^ It is a reliable and valid assessment of suicidal ideation and behavior when used in both clinical and research settings with young adults^29^ and assesses the frequency and intensity of suicidal ideation, nonsuicidal self-injury, and SB, including interrupted or aborted suicide attempts, preparatory behaviors, and actual suicide attempts. Lifetime history of SB was coded at baseline as a binary variable, with 0 representing no history of SB and 1 indicating actual suicide attempt with clear intent to end their life and any interrupted, aborted, and ambiguous attempts as defined on the C-SSRS at or prior to baseline. The C-SSRS was also administered at follow-up visits at 3, 6, and 12 months after baseline at in-person and online study visits. We also used the LIFE to determine onset and/or recurrence of SB using the C-SSRS domains. In addition to the C-SSRS and LIFE to track SB prospectively, all patients were followed up in the EHR to track ED visits and hospitalization for SB and SB reported in outpatient settings. SB was identified using an *International Statistical Classification of Diseases and Related Health Problems, Tenth Revision *(*ICD-10*) diagnostic code entered by clinicians on encounters (T14.91). We also reviewed EHR notes to identify SB. While 53 patients (31%) had follow-up study visits, information on SB during the study period was assessed for all participants using EHR records, and 133 participants (78%) had at least 1 encounter with the University of Pittsburgh Medical Center (UPMC) EHR during the follow-up period or a follow-up visit. There were no significant associations between primary variables of interest and retention at follow-up visits or retention overall (eTables 1 and 2 in Supplement 1).

#### Self-Reported Symptoms and Factors Associated With Increased and Decreased Risk

Self-report questionnaires assessed the severity of psychiatric symptoms and factors associated with increased and decreased risk. These included depressive symptoms with the Patient Health Questionnaire,^30^ anxiety symptoms with the Generalized Anxiety Disorder-7 scale,^31^ Beck Hopelessness Scale,^32^ Barratt Impulsiveness Scale,^33^ Adult Suicidal Ideation Questionnaire,^34^ Buss-Perry Aggression Questionnaire,^35^ Reasons for Living,^36^ Perceived Stress Scale,^37^ Affect Lability Scales,^38^ Apathy Evaluation Scale,^39^ anhedonia using Snaith Hamilton Pleasure Scale,^40^ PTSD Checklist for *DSM-5*,^41^ and Multidimensional Scale of Perceived Social Support.^42^ The Drug Use Screening Inventory^43^ and Tobacco Use Questionnaire^44^ measured participants past and current substance use, and the Childhood Trauma Questionnaire^45^ assessed childhood history of abuse and neglect.

### Statistical Analysis

We conducted analyses comparing SM and GM patients with heterosexual and cisgender patients, respectively, on risk for SB in the year after hospital discharge. Because nearly all GM patients also identified as SM, separate analyses were conducted for SM and GM patients. Separate SM and GM comparisons with heterosexual and cisgender participants, respectively, were examined, and effect sizes for each difference are presented (eTables 3 and 4 in Supplement 1). Kaplan-Meier curves were estimated to examine the risk of SB over time between SM and heterosexual patients and between GM and cisgender patients, and log-rank tests were used to determine whether the risk of SB differed between these groups. To assess whether risk for SB differs between SM and heterosexual and between GM and cisgender patients after adjusting for other factors, multivariable Cox proportional hazards (PH) models were estimated. Potential factors associated with SB were screened using bivariable Cox PH modeling. We modeled both SM and GM using a multivariable approach, including demographic characteristics regardless of bivariable significance in each model along with both known factors associated with increased risk for SB (severity of suicidal ideation and lifetime SB) as well as variables that were associated with SB at follow-up at *P* < .10. We used forward and backward variable selection methods to build the most parsimonious model. The Cox PH assumption was established at each modeling step by assessing whether the slope of β for each factor remained constant across time. If PH assumptions were not met, stratified Cox PH models were estimated, and a Firth correction was applied using R package coxphf, where we experienced a monotoned likelihood with lifetime SB included in the model.^46,47^ We conducted power analysis to determine power in each model when including covariates: for a 2-sided test at *P* = .05, we have 80% power to detect a hazard ratio (HR) of 3.0 between the SM and heterosexual groups, and we have 80% power to detect an HR of 5.6 between the GM and cisgender groups. Additionally, we found the risk for SB was highest within approximately 3 months following discharge after inspection of the Kaplan-Meier curves, as the highest proportion of events occurred within 100 days and Kaplan-Meier curves evidenced the steepest slope within 100 days. Given this pattern aligns with prior studies and because initial GM multivariable models did not meet the Cox PH assumption for the follow-up period,^2^ additional sensitivity analyses for both SM and GM models were conducted in which SB events after 100 days were censored, and any time to event greater than 100 days was censored at 100 days. Low missingness was observed for variables included in multivariable models (0-16% across variables), and our multivariable models used complete-case analysis. Analyses were conducted in R Studio version 2022.07.1 (R Project for Statistical Computing).

## Results

The median (IQR) age of the 160 patients included was 23.5 (IQR, 20.4-27.6) years, 77 (48%) reported male sex assigned at birth, and 114 (71%) identified their race as White. Demographic and clinical characteristics among SM and GM participants, compared with heterosexual and cisgender participants, are presented in eTables 3 and 4 in Supplement 1. SM and GM participants reported higher suicidal ideation severity at baseline. While SM participants were more likely to report a lifetime history of SB than heterosexual participants, history of SB did not differ between GM and cisgender participants.

**Figure 1.**
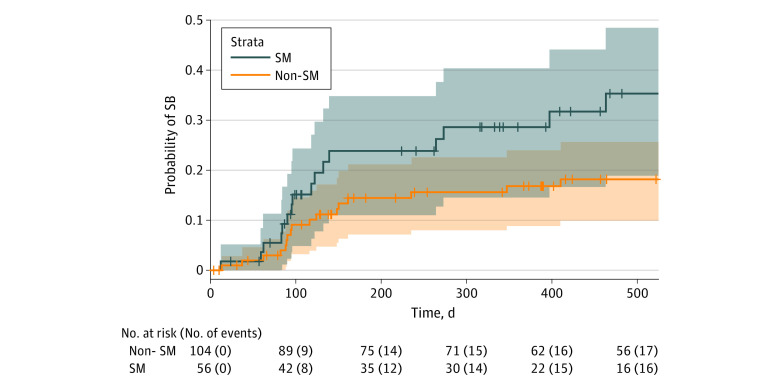
Kaplan-Meier Curves for Suicidal Behavior (SB) Over Time Among Sexual Minority (SM) and Non-SM Patients

**Figure 2.**
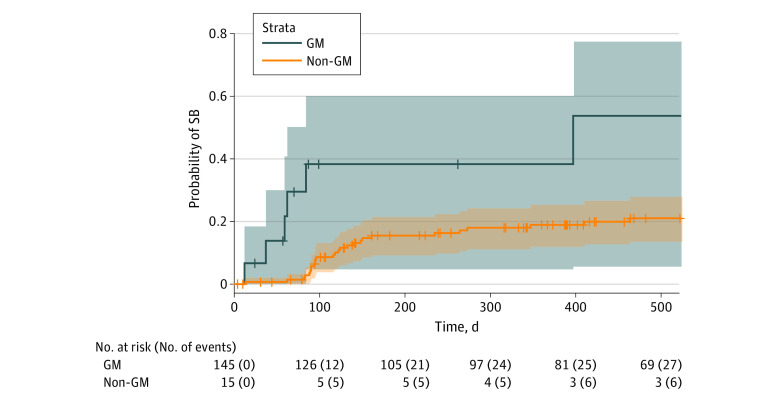
Kaplan-Meier Curves for Suicidal Behavior (SB) Over Time Among Gender Minority (GM) and Non-GM Patients

During the follow-up period, 33 SB events occurred (21% of patients). The proportions of SM and GM patients that experienced SB at follow-up was 29% (16 patients) and 40% (6 patients), respectively. Separate Kaplan-Meier curves indicated SM (HR, 2.02; 95% CI, 1.02-4.00; log-rank *P* = .04) and GM (HR, 4.27; 95% CI, 1.75-10.40; log-rank *P* < .001) patients had significantly higher risk for SB compared with their heterosexual and cisgender counterparts, respectively, in bivariable analyses (Figures 1 and 2; Tables 1 and 2). Sensitivity analyses were conducted on events from baseline to 100 days, as we found that 17 events (52%) happened within the first 100 days after baseline. In sensitivity analyses, differences in risk for SB between SM and heterosexual patients were no longer significant after censoring events greater than 100 days (HR, 1.71; 95% CI, 0.66–4.44; log-rank *P* = .27) (eTable 5 in Supplement 1). However, differences in risk for SB between GM and cisgender patients persisted when examining only 100 days following discharge (HR, 6.90; 95% CI, 2.41-19.78; log-rank *P* < .001) (Table 3).

**Table 1.** Proportional Hazards Models Examining Suicidal Behavior Among Sexual Minority and Non–Sexual Minority Patients

Model and term	HR (95% CI)	*P* value
No adjustments		
Sexual minority vs non–sexual minority	2.02 (1.02-4.00)	.04
Adjusting for demographics		
Sexual minority vs non–sexual minority	2.10 (0.97-4.53)	.06
Race, White vs minorized racial and ethnic group	1.15 (0.53-2.49)	.73
Age, y	0.98 (0.80-1.07)	.63
Sex at birth, male vs female	1.06 (0.49-2.39)	.88
Adjusting for clinical characteristics		
Sexual minority vs non–sexual minority	1.01 (0.47-2.18)	.98
Tobacco use, current vs not	0.28 (0.11-0.66)	.004
PTSD diagnosis, current vs not	3.06 (1.40-6.65)	.01
Suicidal ideation	1.01 (1.00-1.02)	.16
Apathy	1.05 (1.00-1.11)	.07
Suicidal behavior, lifetime vs none	2.22 (0.27-18.32)	.46

Abbreviations: HR, hazard ratio; PTSD, posttraumatic stress disorder.

**Table 2.** Proportional Hazards Models Examining Suicidal Behavior Among Gender Minority and Non–Gender Minority Patients

Model and term^a^	HR (95% CI)	*P* value
No adjustments		
Gender minority vs non–gender minority	4.27 (1.75-10.40)	.001
Adjusting for demographic characteristics		
Gender minority vs non–gender minority	4.85 (1.90-12.38)	.001
Age, y	1.00 (0.92-1.10)	.93
Adjusting for clinical characteristics		
Gender minority vs non–gender minority	2.18 (0.73-5.61)	.15
Tobacco use, current vs not	0.30 (0.12-0.70)	.004
PTSD diagnosis, current vs not	2.99 (1.42-6.41)	.004
Suicidal ideation	1.01 (1.00-1.02)	.12
Suicidal behavior, lifetime vs none	1.65 (0.35-16.07)	.56

Abbreviations: HR, hazard ratio; PTSD, posttraumatic stress disorder.

^a^ Final model adjusting for demographic characteristics using a stratified Cox proportional hazard model on race. Final model adjusting for clinical characteristics using a Firth correction.

**Table 3.** Gender Minority Models Censoring Events Greater than 100 Days

Model and term^a^	HR (95% CI)	*P* value
No adjustments		
Gender minority vs non–gender minority	6.90 (2.41-19.78)	<.001
Adjusting for demographics		
Gender minority vs non–gender minority	8.75 (2.83-27.11)	<.001
Age, y	0.96 (0.85-1.09)	.55
Adjusting for clinical characteristics		
Gender minority vs non–gender minority	3.80 (1.18-11.19)	.03
PTSD diagnosis, current vs not	3.49 (1.30-10.05)	.01
Tobacco use, current vs not	0.28 (0.07-0.88)	.03
Suicide ideation	1.01 (1.00-1.03)	.16
Suicidal behavior, lifetime vs none	2.23 (0.23-301.56)	.56

Abbreviations: HR, hazard ratio; PTSD, posttraumatic stress disorder.

^a^ Final model adjusting for demographic characteristics using a stratified Cox proportional hazard model on race. Final model adjusting for clinical characteristics using a Firth correction.

Multivariable Cox PH models were estimated to examine risk for SB in SM and GM groups compared with heterosexual and cisgender groups, while controlling for demographic characteristics and other factors associated with SB. Clinical characteristics associated with risk of SB at the bivariable level included in the multivariable modeling approach were current PTSD diagnosis, current tobacco use, apathy, reasons for living, suicidal ideation, social support, and anhedonia (eTable 6 in Supplement 1). SM patients did not exhibit elevated risk for SB after adjusting for demographic characteristics (age, sex, and race: HR, 2.10; 95% CI, 0.97-4.53; *P* = .06) and after adjusting for clinical characteristics (HR, 1.01; 95% CI, 0.47-2.18; *P* = .98) (Table 1), and these results persisted within sensitivity analyses conducted while removing individuals who endorsed “Not sure” on the sexual orientation item (HR, 0.73; 95% CI, 0.30-1.76; *P* = .48). However, GM patients evidenced higher risk for SB (HR, 4.85; 95% CI, 1.90-12.38, *P* = .001) after adjusting for demographic characteristics (Table 2). Adjusting for clinical characteristics revealed no difference in risk for SB between GM and cisgender participants when examining the full follow-up period (HR, 2.18; 95% CI, 0.73-5.61; *P* = .15) (Table 2). Similar results were observed for SM compared with heterosexual patients in sensitivity analyses censoring events after 100 days (eTable 5 in Supplement 1). However, GM patients evidenced higher risk for SB compared with cisgender patients when censoring events after 100 days and after adjusting for demographic and clinical characteristics (HR, 3.80; 95% CI, 1.18-11.19; *P* = .03) (Table 3).

## Discussion

We found higher rates of SB following inpatient psychiatric hospitalization among both SM and GM patients compared with other psychiatric inpatients who identified as heterosexual and cisgender. However, this risk was no longer significant when controlling for demographic and clinical characteristics, suggesting that elevated rates of SB among SM and GM patients following discharge are likely explained by clinical characteristics at admission. However, GM patients showed higher rates of SB during the 100 days following discharge even after controlling for clinical characteristics associated with risk for suicidal behavior.

While SGM individuals have documented elevated risk for psychopathology and suicidal behavior, these populations also experience numerous barriers to receiving mental health services^48,49^ that could contribute to their elevated risk for suicidal behavior following discharge from psychiatric hospitalization. SGM individuals also experience discrimination and stigmatization within mental health care settings.^50,51,52,53^ Furthermore, it could be difficult for SGM individuals to find clinicians who have sufficient training to provide services tailored to the needs and experiences of SGM patients,^54^ so SGM individuals might be more likely to fall out of the continuum of care following their discharge. Discrimination and invalidation in health care settings could be particularly problematic among GM individuals,^51,52^ contributing to their elevated risk for suicidal behavior observed in the current study. While we did not measure the unique risk factors that could disproportionately affect GM individuals,^51,52,55,56^ emerging evidence indicates discriminatory health care encounters could contribute to their risk for suicidal behavior.^56^ Furthermore, GM individuals are less likely to have health insurance compared with cisgender individuals and might avoid care if they have experienced discrimination in health care settings.^57^ These barriers could make it especially difficult for GM individuals to obtain the required outpatient care following hospitalization. Furthermore, the intersection of racial and ethnic minority identities and GM status could be related to more severe barriers to receiving mental health services among GM patients who belong to minoritized racial and ethnic groups.^58,59^ Future studies should investigate the experiences of diverse GM individuals during and following inpatient hospitalization to determine which factors contribute to their increased risk for suicidal behavior during this pivotal period of time.

Notably, SGM patients were overrepresented within our sample of psychiatric inpatients. More than one-third of the sample identified as SM (35%), and 9% of the sample identified as GM. While it is difficult to ascertain accurate estimates of the proportion of SGM individuals in the United States because they are hidden populations, recent census data indicate 10% of individuals identify as SGM, with 1% of the population identifying as GM.^60^ The disproportionate representation of SGM individuals in our data are similar to that observed in another study of SGM youth receiving ED services,^23^ and this pattern is consistent with higher rates of psychopathology and SB within SGM populations.

As part of the risk assessment and monitoring protocols on inpatient psychiatric units, it is critical to assess sexual orientation and gender identity among all patients, and clinicians should attend to how the unique minority stress experiences of SGM individuals contribute to their psychiatric symptoms.^17,19,20,61^ Clinicians and all staff who interact with patients must use GM individuals’ affirmed name and pronouns.^62^ Clinical care and discharge plans should be tailored to the needs of SGM individuals, including referrals to outpatient clinicians who have the requisite training and competence to provide validating care to SGM individuals.^63^ To facilitate this care for SGM individuals during and following inpatient hospitalization, systemic changes are required to increase the amount of education and training clinicians receive to reduce biases toward SGM individuals and increase knowledge and competence with SGM populations.^64,65^

### Strengths and Limitations

The contributions of the present study must be understood within the context of its strengths and limitations. Our sample included diverse psychiatric patients across the spectrum of psychopathology. Retention of psychiatric patients in longitudinal studies is challenging given the severity of their symptoms, and our retention rate for follow-up visits was similar to the rates at which patients keep their outpatient appointments following hospitalization.^66,67^ Our use of UPMC EHR data from both inpatient and outpatient settings to track SB events for all patients over time following discharge is a notable strength. We collected both structured and unstructured EHR data and did not rely on SB being explicitly coded as a diagnosis. However, this approach cannot document SB reported to clinicians outside the UPMC system or not reported to clinicians. SGM patients could be less likely to seek medical care for future suicide attempts or report these to clinicians, especially if they experienced stigmatization during their care, potentially attenuating observed differences in risk for suicidal behavior between SGM and other patients. Furthermore, the GM group was small, and thus our power to detect significant differences is limited. In addition, nearly all SB events among GM patients occurred within the first 100 days following discharge (5 of 6 events). However, the effect size for the difference between GM and cisgender patients during the first 100 days was sufficiently large that we were able to detect it despite limited power. While this result could suggest disproportionate risk for suicidal behavior among GM patients during the 100 days following discharge in particular, future studies with larger samples of GM patients are needed to examine differences in risk during a longer follow-up period. Additionally, we had to exclude participants from the present analysis if they did not have information on sexual orientation and gender identity, and included participants were more likely to have anxiety and PTSD diagnoses. We have included these variables as covariates in our analytic approach to minimize any potential biases related to being included in the present analysis.

## Conclusions

In this cohort study of psychiatric inpatients, SGM patients had elevated risk for suicidal behavior following discharge, and this risk was not accounted for by clinical characteristics at admission among GM patients during the 100 days after discharge. Given the disproportionate representation of SGM individuals in the inpatient population, as well as their increased risk for suicidal behavior following discharge, additional attention to validating and effective care for SGM individuals within inpatient psychiatric settings is warranted.

## Notes

Supplement 1. **eTable 1.** Associations Between Demographic and Clinical Characteristics and Retention at Follow-Up Visits **eTable 2.** Associations Between Demographic and Clinical Characteristics and Retention at Follow-Up Visits or Within Electronic Health Records **eTable 3.** Demographic and Clinical Characteristics Between Sexual Minority and Non-Sexual Minority Patients **eTable 4.** Demographic and Clinical Characteristics Between Gender Minority and Non-Gender Minority Patients **eTable 5.** Sexual Minority Models Censoring Events Greater Than 100 Days **eTable 6.** Associations Between Demographic and Clinical Characteristics and Suicidal Behavior During Follow-Up Period

Supplement 2.
Data Sharing Statement
