# Supplementary material for: Risk for Suicidal Behavior After Psychiatric Hospitalization Among Sexual and Gender Minority Patients
Source: JAMA Netw Open. 2023 Sep 8;6(9):e2333060. doi: 10.1001/jamanetworkopen.2023.33060 (PMC10492186; doi:10.1001/jamanetworkopen.2023.33060)
Supplement: Supplement 1. — eTable 1. Associations Between Demographic and Clinical Characteristics and Retention at Follow-Up Visits eTable 2. Associations Between Demographic and Clinical Characteristics and Retention at Follow-Up Visits or Within Electronic Health Records eTable 3. Demographic and Clinical Characteristics Between Sexual Minority and Non-Sexual Minority Patients eTable 4. Demographic and Clinical Characteristics Between Gender Minority and Non-Gender Minority Patients eTable 5. Sexual Minority Models Censoring Events Greater Than 100 Days eTable 6. Associations Between Demographic and Clinical Characteristics and Suicidal Behavior During Follow-Up Period [file jamanetwopen-e2333060-s001.pdf]

Supplemental Online Content

Thoma BC, Hone E, Roig A, et al. Risk for suicidal behavior after psychiatric hospitalization among sexual and gender minority young adults. *JAMA Netw Open.* 2023;6(9):e2333060. doi:10.1001/jamanetworkopen.2023.33060

eTable 1. Associations Between Demographic and Clinical Characteristics and Retention at Follow-Up Visits

eTable 2. Associations Between Demographic and Clinical Characteristics and Retention at Follow-Up Visits or Within Electronic Health Records

eTable 3. Demographic and Clinical Characteristics Between Sexual Minority and Non-Sexual Minority Patients

eTable 4. Demographic and Clinical Characteristics Between Gender Minority and Non-Gender Minority Patients

eTable 5. Sexual Minority Models Censoring Events Greater Than 100 Days

eTable 6. Associations Between Demographic and Clinical Characteristics and Suicidal Behavior During Follow-Up Period

This supplemental material has been provided by the authors to give readers additional information about their work.

© 2023 Thoma BC et al. *JAMA Network Open.*

eTable 1. Associations Between Demographic and Clinical Characteristics and Retention at Follow-Up Visits

Term	[ALL][a] N=160	N	No Follow-up[a] N=107	Follow-up[a] N=53	p-value[b]	Effect Size[c]	Cohen's d
Demographics and Sample Characteristics							
Age (years)	23.5 [20.4;27.6]	160	23.1 [20.6;27.3]	24.1 [20.4;28.7]	0.49	-0.003	-0.13
Body mass index	24.8 [21.0;30.1]	106	24.0 [21.0;27.7]	25.7 [20.9;32.3]	0.21	0.004	-0.29
Sex at birth, Male	77 (48.1%)	160	53 (49.5%)	24 (45.3%)	0.74	0.84	-
Race, White	114 (71.2%)	160	74 (69.2%)	40 (75.5%)	0.52	1.37	-
Gender minority	15 (9.38%)	160	7 (6.54%)	8 (15.1%)	0.14	2.54	-
Sexual minority	56 (35.0%)	160	35 (32.7%)	21 (39.6%)	0.49	0.74	-
Gender affirming hormone therapies	5 (3.57%)	140	3 (3.33%)	2 (4.00%)	>0.99	1.21	-
Psychiatric Disorders, Current							
Bipolar disorder	25 (15.7%)	159	14 (13.1%)	11 (21.2%)	0.28	1.78	-
Unipolar diagnosis	131 (82.4%)	159	86 (80.4%)	45 (86.5%)	0.46	1.57	-
Bipolar disorder or Unipolar diagnosis	141 (88.7%)	159	93 (86.9%)	48 (92.3%)	0.43	1.81	-
Psychotic disorder	14 (8.81%)	159	9 (8.41%)	5 (9.62%)	0.77	1.16	-
Alcohol use disorder	68 (42.8%)	159	46 (43.0%)	22 (42.3%)	>0.99	0.97	-
Substance use disorder	66 (41.5%)	159	42 (39.3%)	24 (46.2%)	0.51	1.33	-
Alcohol use disorder or Substance use disorder	90 (56.6%)	159	61 (57.0%)	29 (55.8%)	>0.99	0.95	-
Anxiety disorders	68 (42.8%)	159	35 (32.7%)	33 (63.5%)	<0.001	3.57	-
Post-traumatic stress disorder diagnosis	49 (30.8%)	159	25 (23.4%)	24 (46.2%)	0.01	2.81	-
Substance Use							
Substance use severity score, past year	40.0 [6.67;73.3]	153	40.0 [13.3;80.0]	23.3 [1.67;60.0]	0.08	0.01	0.27
Tobacco use, current	64 (47.8%)	134	47 (53.4%)	17 (37.0%)	0.10	0.51	-
Clinical Characteristics							
Anxiety	13.0 [8.00;16.0]	157	13.0 [8.00;16.0]	12.5 [7.00;16.0]	0.78	-0.006	0.05
Depression	18.0 [12.0;21.0]	157	17.0 [12.0;21.0]	18.5 [12.0;22.0]	0.43	-0.002	-0.14
Hopelessness	10.0 [9.00;12.0]	157	10.0 [9.00;12.0]	10.0 [9.00;11.0]	0.62	-0.01	0.04
Physical abuse	7.00 [5.00;11.0]	156	7.00 [5.00;11.0]	7.00 [5.00;11.0]	0.97	-0.01	0.05
Sexual abuse	5.00 [5.00;12.5]	155	5.00 [5.00;12.2]	5.00 [5.00;12.0]	0.30	0.001	-0.08
Childhood trauma questionnaire total	59.0 [53.0;67.2]	156	59.0 [53.0;67.2]	57.5 [53.0;67.2]	0.70	-0.01	0.07
Post-traumatic stress disorder symptoms	42.0 [26.0;56.0]	149	41.0 [30.2;55.5]	45.0 [25.0;55.5]	0.63	-0.01	-0.08
Aggression*	0.54 (0.16)	153	0.55 (0.15)	0.53 (0.16)	0.58	0.10	-
Affective lability	1.80 [1.22;2.15]	153	1.80 [1.26;2.15]	1.85 [1.14;2.18]	0.89	-0.01	-0.01
Apathy*	42.2 (7.89)	139	41.8 (7.95)	42.9 (7.80)	0.47	-0.13	-
Reasons for living	162 [131;197]	150	171 [133;208]	158 [124;185]	0.14	0.01	0.29
Suicide ideation	86.0 [43.0;113]	157	83.0 [36.0;111]	96.5 [54.8;116]	0.10	0.01	-0.28
Impulsivity*	71.7 (12.5)	134	72.2 (12.2)	70.7 (13.0)	0.51	0.12	-
Social support	5.21 [4.10;5.92]	138	5.29 [4.44;6.00]	4.46 [3.25;5.77]	0.01	0.04	0.55

© 2023 Thoma BC et al. *JAMA Network Open.*

Term	[ALL][a] N=160	N	No Follow-up[a] N=107	Follow-up[a] N=53	p-value[b]	Effect Size[c]	Cohen's d
Perceived stress	28.0 [24.0;31.0]	136	28.0 [22.0;31.0]	28.0 [25.0;31.0]	0.28	0.001	-0.31
Anhedonia	3.50 [1.00;5.00]	136	3.00 [1.00;5.00]	4.00 [2.00;6.25]	0.09	0.01	-0.33
Symptomatology	106 [83.0;128]	150	106 [83.5;127]	103 [82.8;128]	0.86	-0.01	0.03
Lifetime History							
Suicidal behavior	137 (86.2%)	159	89 (83.2%)	48 (92.3%)	0.15	2.43	-

[a] Mean (SD) for normal, continuous (*); Median [IQR] for skewed, continuous; N (%) for categorical

[b] t-test for normal continuous (*), Kruskal – Wallis test for skewed, continuous; Chi-squared or Fisher's exact test where appropriate for categorical

[c] Cohen's D for normal (*), continuous; η^2 for skewed continuous; Odds Ratio for categorical

© 2023 Thoma BC et al. *JAMA Network Open.*

eTable 2. Associations Between Demographic and Clinical Characteristics and Retention at Follow-Up Visits or Within Electronic Health Records

Term	[ALL][a] N=160	N	No Follow-up[a] N=27	Follow-up[a] N=133	p-value[b]	Effect Size[c]	Cohen's d
Age (years)	23.5 [20.4;27.6]	160	23.1 [20.2;26.1]	23.6 [20.5;27.8]	0.43	-0.002	-0.17
Body mass index	24.8 [21.0;30.1]	106	23.4 [21.1;27.4]	25.1 [21.0;30.8]	0.54	-0.004	-0.08
Sex at birth, Male	77 (48.1%)	160	12 (44.4%)	65 (48.9%)	0.84	1.19	-
Race, White	114 (71.2%)	160	22 (81.5%)	92 (69.2%)	0.25	0.51	-
Gender minority	15 (9.38%)	160	2 (7.41%)	13 (9.77%)	>0.99	1.35	-
Sexual minority	56 (35.0%)	160	9 (33.3%)	47 (35.3%)	>0.99	0.92	-
Gender affirming hormone therapies	5 (3.57%)	140	2 (9.09%)	3 (2.54%)	0.18	0.26	-
Psychiatric Disorders, Current							
Bipolar disorder	25 (15.7%)	159	6 (22.2%)	19 (14.4%)	0.47	0.59	-
Unipolar diagnosis	131 (82.4%)	159	20 (74.1%)	111 (84.1%)	0.33	1.85	-
Bipolar or Unipolar diagnosis	141 (88.7%)	159	24 (88.9%)	117 (88.6%)	>0.99	0.97	-
Psychotic disorder	14 (8.81%)	159	1 (3.70%)	13 (9.85%)	0.47	2.84	-
Alcohol use disorder	68 (42.8%)	159	12 (44.4%)	56 (42.4%)	>0.99	0.92	-
Substance use disorder	66 (41.5%)	159	10 (37.0%)	56 (42.4%)	0.76	1.25	-
Alcohol use disorder or Substance use disorder	90 (56.6%)	159	15 (55.6%)	75 (56.8%)	>0.99	1.05	-
Anxiety disorders	68 (42.8%)	159	11 (40.7%)	57 (43.2%)	0.98	1.11	-
Post-traumatic stress disorder diagnosis	49 (30.8%)	159	4 (14.8%)	45 (34.1%)	0.07	2.97	-
Substance Use							
Substance use severity score, past year	40.0 [6.67;73.3]	153	20.0 [13.3;66.7]	40.0 [6.67;73.3]	0.59	-0.005	-0.15
Tobacco use, current	64 (47.8%)	134	11 (45.8%)	53 (48.2%)	>0.99	1.10	-
Clinical Characteristics							
Anxiety	13.0 [8.00;16.0]	157	14.0 [10.0;16.5]	12.5 [7.00;16.0]	0.37	-0.001	0.17
Depression	18.0 [12.0;21.0]	157	19.0 [12.5;22.5]	17.0 [12.0;21.0]	0.37	-0.001	0.15
Hopelessness	10.0 [9.00;12.0]	157	10.0 [8.00;11.5]	10.5 [9.00;12.0]	0.46	-0.003	-0.13
Physical abuse	7.00 [5.00;11.0]	156	6.00 [5.00;7.50]	7.00 [5.00;11.0]	0.04	0.020	-0.44
Sexual abuse	5.00 [5.00;12.5]	155	5.00 [5.00;7.00]	5.00 [5.00;13.0]	0.17	0.006	-0.24
Childhood trauma questionnaire total	59.0 [53.0;67.2]	156	55.0 [52.0;62.5]	59.0 [53.0;68.0]	0.10	0.011	-0.36
Post-traumatic stress disorder symptoms	42.0 [26.0;56.0]	149	37.0 [28.0;48.0]	44.0 [26.2;56.8]	0.32	0.00005	-0.21
Aggression*	0.54 (0.16)	153	0.53 (0.13)	0.55 (0.16)	0.52	-0.122	-
Affective lability	1.80 [1.22;2.15]	153	1.80 [1.26;2.21]	1.81 [1.19;2.15]	0.83	-0.006	0.08
Apathy*	42.2 (7.89)	139	41.0 (6.93)	42.4 (8.08)	0.39	-0.176	-
Reasons for living	162 [131;197]	150	165 [131;193]	161 [131;199]	0.93	-0.006	-0.01
Suicide ideation	86.0 [43.0;113]	157	83.0 [23.5;110]	86.5 [47.0;113]	0.43	-0.002	-0.18
Impulsivity*	71.7 (12.5)	134	73.2 (12.4)	71.4 (12.5)	0.53	0.145	-
Social support	5.21 [4.10;5.92]	138	5.50 [4.48;5.94]	5.08 [3.94;5.83]	0.11	0.010	0.42
Perceived stress	28.0 [24.0;31.0]	136	27.0 [21.8;31.0]	28.0 [24.0;31.0]	0.59	-0.004	-0.24
Anhedonia	3.50 [1.00;5.00]	136	4.00 [0.00;4.25]	3.00 [1.00;5.25]	0.37	-0.001	-0.26

© 2023 Thoma BC et al. *JAMA Network Open.*

Term	[ALL][a] N=160	N	No Follow-up[a] N=27	Follow-up[a] N=133	p-value[b]	Effect Size[c]	Cohen's d
Symptomatology	106 [83.0;128]	150	107 [90.0;126]	105 [83.0;128]	0.75	-0.006	0.04
Lifetime History							
Suicidal behavior	137 (86.2%)	159	22 (81.5%)	115 (87.1%)	0.54	1.54	-

[a] Mean (SD) for normal, continuous (*); Median [IQR] for skewed, continuous; N (%) for categorical

[b] t-test for normal continuous (*), Kruskal – Wallis test for skewed, continuous; Chi-squared or Fisher's exact test where appropriate for categorical

[c] Cohen's D for normal (*), continuous; η^2 for skewed continuous; Odds Ratio for categorical

© 2023 Thoma BC et al. *JAMA Network Open.*

eTable 3. Demographic and Clinical Characteristics Between Sexual Minority and Non-Sexual Minority Patients

Term	[ALL][a] N=160	N	SM[a] N=56	Non – SM[a] N=104	p-value[b]	Effect Size[c]	Cohen's d
Demographics and Sample Characteristics							
Sex at birth, Male	77 (48.1%)	160	12 (21.4%)	65 (62.5%)	<0.001	0.16	-
Race, White	114 (71.2%)	160	39 (69.6%)	75 (72.1%)	0.88	0.89	-
Age (years)	23.5 [20.4;27.6]	160	23.6 [20.5;27.4]	23.3 [20.2;27.6]	0.99	-0.01	-0.01
Body mass index	24.8 [21.0;30.1]	106	29.1 [21.5;35.5]	23.6 [20.9;26.9]	0.01	0.03	-0.74
Gender minority	15 (9.38%)	160	14 (25.0%)	1 (0.96%)	<0.001	34.33	-
Gender affirming hormone therapies	5 (3.57%)	140	5 (10.4%)	0 (0.00%)	-	-	-
Psychiatric Disorders, Current							
Bipolar disorder	25 (15.7%)	159	14 (25.0%)	11 (10.7%)	0.03	2.79	-
Unipolar diagnosis	131 (82.4%)	159	45 (80.4%)	86 (83.5%)	0.78	0.81	-
Bipolar disorder or Unipolar diagnosis	141 (88.7%)	159	51 (91.1%)	90 (87.4%)	0.61	1.47	-
Psychotic disorder	14 (8.81%)	159	3 (5.36%)	11 (10.7%)	0.38	0.47	-
Alcohol use disorder	68 (42.8%)	159	20 (35.7%)	48 (46.6%)	0.25	0.64	-
Substance use disorder	66 (41.5%)	159	19 (33.9%)	47 (45.6%)	0.21	0.61	-
Alcohol use disorder or Substance use disorder	90 (56.6%)	159	27 (48.2%)	63 (61.2%)	0.16	0.59	-
Anxiety disorders	68 (42.8%)	159	25 (44.6%)	43 (41.7%)	0.85	1.13	-
Post-traumatic stress disorder diagnosis	49 (30.8%)	159	21 (37.5%)	28 (27.2%)	0.24	1.61	-
Substance Use							
Substance use severity score, past year	40.0 [6.67;73.3]	153	23.3 [6.67;66.7]	40.0 [13.3;73.3]	0.15	0.01	0.24
Tobacco use, current	64 (47.8%)	134	21 (44.7%)	43 (49.4%)	0.73	0.83	-
Clinical Characteristics							
Anxiety	13.0 [8.00;16.0]	157	13.0 [10.0;16.8]	12.0 [7.50;16.0]	0.49	-0.003	-0.13
Depression	18.0 [12.0;21.0]	157	19.0 [15.0;22.8]	17.0 [10.5;20.0]	0.02	0.03	-0.42
Hopelessness	10.0 [9.00;12.0]	157	11.0 [8.25;12.0]	10.0 [9.00;11.5]	0.44	-0.003	-0.09
Physical abuse	7.00 [5.00;11.0]	156	8.00 [5.00;13.0]	7.00 [5.00;10.5]	0.16	0.01	-0.2
Sexual abuse	5.00 [5.00;12.5]	155	6.00 [5.00;16.0]	5.00 [5.00;7.75]	0.01	0.04	-0.45
Childhood trauma questionnaire total	59.0 [53.0;67.2]	156	62.0 [54.0;72.0]	57.0 [53.0;64.0]	0.04	0.02	-0.33
Post-traumatic stress disorder symptoms	42.0 [26.0;56.0]	149	48.0 [37.0;57.2]	38.0 [23.0;54.0]	0.003	0.05	-0.5
Aggression*	0.54 (0.16)	153	0.56 (0.15)	0.53 (0.16)	0.30	-0.18	-0.18
Affective lability	1.80 [1.22;2.15]	153	1.88 [1.49;2.32]	1.69 [1.09;2.07]	0.06	0.02	-0.35
Apathy*	42.2 (7.89)	139	44.7 (7.72)	40.8 (7.68)	0.01	-0.50	-0.50
Reasons for living	162 [131;197]	150	140 [120;184]	174 [136;207]	0.004	0.05	0.53
Suicide ideation	86.0 [43.0;113]	157	104 [71.2;118]	80.0 [30.0;110]	0.001	0.06	-0.57
Impulsivity*	71.7 (12.5)	134	74.8 (12.4)	70.0 (12.3)	0.04	-0.39	-0.39
Social support	5.21 [4.10;5.92]	138	4.71 [4.15;5.58]	5.37 [4.10;5.98]	0.10	0.01	0.24
Perceived stress	28.0 [24.0;31.0]	136	28.0 [25.8;31.0]	27.0 [22.8;31.0]	0.25	0.002	-0.23
Anhedonia	3.50 [1.00;5.00]	136	4.00 [2.00;6.00]	3.00 [1.00;5.00]	0.26	0.002	-0.23

© 2023 Thoma BC et al. *JAMA Network Open.*

Symptomatology	106 [83.0;128]	150	115 [94.5;136]	102 [76.0;118]	0.01	0.04	-0.50
Lifetime History							
Suicidal behavior	137 (86.2%)	159	52 (92.9%)	85 (82.5%)	0.09	2.75	-
Outcome							
Suicidal behavior, after baseline	33 (20.6%)	160	16 (28.6%)	17 (16.3%)	0.11	2.05	-

[a]Mean (SD) for normal, continuous (*); Median [IQR] for skewed, continuous; N (%) for categorical

[b]t-test for normal continuous (*), Kruskal – Wallis test for skewed, continuous; Chi-squared or Fisher's exact test where appropriate for categorical

[c]Cohen's D for normal (*), continuous; η^2 for skewed continuous; Odds Ratio for categorical

© 2023 Thoma BC et al. *JAMA Network Open.*

eTable 4. Demographic and Clinical Characteristics Between Gender Minority and Non-Gender Minority Patients

Term	[ALL][a] N=160	N	GM[a] N=15	Non-GM[a] N=145	p-value[b]	Effect Size[c]	Cohen's d
Demographics and Sample Characteristics							
Sex at birth, Male	77 (48.1%)	160	4 (26.7%)	73 (50.3%)	0.1	0.36	-
Race, White	114 (71.2%)	160	9 (60.0%)	105 (72.4%)	0.4	0.57	-
Age (years)	23.5 [20.4;27.6]	160	23.2 [20.0;26.3]	23.6 [20.5;27.8]	0.4	-0.003	0.21
Body mass index	24.8 [21.0;30.1]	106	30.7 [20.9;39.7]	24.5 [21.0;29.4]	0.3	-0.0007	-0.98
Gender minority	56 (35.0%)	160	14 (93.3%)	42 (29.0%)	<0.001	34.33	-
Gender affirming hormone therapies	5 (3.57%)	140	5 (33.3%)	0 (0.00%)	<0.001	-	-
Psychiatric Disorders, Current							
Bipolar disorder	25 (15.7%)	159	3 (20.0%)	22 (15.3%)	0.71	1.39	-
Unipolar diagnosis	131 (82.4%)	159	15 (100%)	116 (80.6%)	*0.08*	-	-
Bipolar disorder or Unipolar diagnosis	141 (88.7%)	159	15 (100%)	126 (87.5%)	0.22	-	-
Psychotic disorder	14 (8.81%)	159	0 (0.00%)	14 (9.72%)	0.37	-	-
Alcohol use disorder	68 (42.8%)	159	2 (13.3%)	66 (45.8%)	0.03	0.18	-
Substance use disorder	66 (41.5%)	159	3 (20.0%)	63 (43.8%)	0.13	0.32	-
Alcohol use disorder or Substance use disorder	90 (56.6%)	159	5 (33.3%)	85 (59.0%)	0.10	0.35	-
Anxiety disorders	68 (42.8%)	159	7 (46.7%)	61 (42.4%)	0.96	1.19	-
Post-traumatic stress disorder diagnosis	49 (30.8%)	159	7 (46.7%)	42 (29.2%)	0.24	2.12	-
Substance Use							
Substance use severity score, past year	40.0 [6.67;73.3]	153	6.67 [0.00;20.0]	40.0 [13.3;73.3]	0.00	0.05	0.83
Tobacco use, current	64 (47.8%)	134	1 (8.33%)	63 (51.6%)	0.01	0.09	-
Clinical Characteristics							
Anxiety	13.0 [8.00;16.0]	157	12.0 [10.0;17.0]	13.0 [7.25;16.0]	0.37	-0.001	-0.27
Depression	18.0 [12.0;21.0]	157	22.0 [20.0;24.5]	17.0 [11.2;20.8]	<0.001	0.07	-0.91
Hopelessness	10.0 [9.00;12.0]	157	11.0 [8.50;12.0]	10.0 [9.00;12.0]	0.70	-0.01	-0.06
Physical abuse	7.00 [5.00;11.0]	156	9.00 [6.00;12.0]	7.00 [5.00;11.0]	0.20	0.004	-0.19
Sexual abuse	5.00 [5.00;12.5]	155	5.00 [5.00;10.0]	5.00 [5.00;13.0]	0.72	-0.01	0.06
Childhood trauma questionnaire total	59.0 [53.0;67.2]	156	61.0 [54.0;68.0]	59.0 [53.0;67.0]	0.90	-0.01	0.08
Post-traumatic stress disorder symptoms	42.0 [26.0;56.0]	149	49.0 [41.0;56.5]	41.0 [26.0;55.0]	*0.09*	0.01	-0.46
Aggression*	0.54 (0.16)	153	0.54 (0.10)	0.54 (0.16)	0.97	-0.09	-0.01
Affective lability	1.80 [1.22;2.15]	153	2.17 [1.23;2.36]	1.80 [1.22;2.11]	0.22	0.003	-0.24
Apathy*	42.2 (7.89)	139	48.5 (7.95)	41.5 (7.61)	0.01	-0.92	-0.92
Reasons for living	162 [131;197]	150	122 [93.5;146]	171 [134;199]	<0.001	0.07	1.06
Suicide ideation	86.0 [43.0;113]	157	114 [103;118]	83.0 [38.0;110]	0.001	0.07	-0.92
Impulsivity*	71.7 (12.5)	134	79.2 (7.56)	70.9 (12.6)	0.002	-0.68	-0.68
Social support	5.21 [4.10;5.92]	138	4.17 [3.75;4.75]	5.25 [4.17;5.92]	0.02	0.03	0.61
Perceived stress	28.0 [24.0;31.0]	136	31.0 [27.0;34.0]	27.0 [23.0;30.5]	0.01	0.03	-0.76
Anhedonia	3.50 [1.00;5.00]	136	4.00 [2.00;10.0]	3.00 [1.00;5.00]	0.13	0.01	-0.6
Symptomatology	106 [83.0;128]	150	119 [106;140]	104 [81.5;126]	0.02	0.03	-0.64

© 2023 Thoma BC et al. *JAMA Network Open.*

Lifetime History							
Suicidal behavior	137 (86.2%)	159	13 (86.7%)	124 (86.1%)	>0.99	1.05	-
Outcome							
Suicidal behavior, after baseline	33 (20.6%)	160	6 (40.0%)	27 (18.6%)	0.09	2.91	-

[a]Mean (SD) for normal, continuous (*); Median [IQR] for skewed, continuous; N (%) for categorical

[b]t-test for normal continuous (*), Kruskal – Wallis test for skewed, continuous; Chi-squared or Fisher's exact test where appropriate for categorical

[c]Cohen's D for normal (*), continuous; η^2 for skewed continuous; Odds Ratio for categorical

© 2023 Thoma BC et al. *JAMA Network Open.*

eTable 5. Sexual Minority Models Censoring Events Greater Than 100 Days

Final Models[a]	Term	HR (95% CI)[b]	p-value
No adjustments	Sexual minority (ref. No)	1.71 (0.66-4.44)	0.27
Adjusting for demographics	Sexual minority (ref. No)	1.61 (0.56-4.61)	0.37
	Race, White (ref. Non-white)	3.33 (0.76-14.69)	0.11
	Age (years)	0.93 (0.82-1.06)	0.26
	Sex at birth, Male (ref. Female)	0.82 (0.28-2.42)	0.72
Adjusting for clinical characteristics[1]	Sexual minority (ref. No)	0.95 (0.34-2.51)	0.91
	Tobacco use, current (ref. No)	0.21 (0.05-0.65)	0.01
	PTSD diagnosis, current (ref. No)	4.08 (1.49-11.99)	0.01
	Suicide ideation	1.01 (1.00-1.03)	0.14
	Apathy	1.08 (1.01-1.17)	0.03
	Suicidal behavior, lifetime (ref. No)	2.08 (0.22-276.82)	0.59

[a]Final model adjusting for demographics using a stratified cox PH model on race. Final model adjusting for clinical characteristics using a Firth correction.
[b]Abbreviations. HR = hazard ratio; CI = confidence interval.

© 2023 Thoma BC et al. *JAMA Network Open.*

eTable 6. Associations Between Demographic and Clinical Characteristics and Suicidal Behavior During Follow-Up Period

Term	HR (95% CI)[a]	p-value
Demographics and Sample Characteristics		
Sex at birth, Male (ref. Female)	0.77 (0.39-1.54)	0.46
Race, White (ref. Non-white)	1.1 (0.51-2.36)	0.82
Age (years)	0.98 (0.90-1.08)	0.72
Body mass index	1.04 (0.98-1.10)	0.18
Sexual minority (ref. No)	2.02 (1.02-4.00)	0.04
Gender minority (ref. No)	4.27 (1.75-10.41)	0.001
Gender affirming hormone therapies (ref. No)	7.78 (2.33-25.97)	0.001
Psychiatric Disorders, Current		
Bipolar disorder (ref. No)	1.85 (0.84-4.11)	0.13
Unipolar diagnosis (ref. No)	1.69 (0.59-4.80)	0.33
Bipolar disorder or Unipolar diagnosis (ref. No)	2.28 (0.55-9.54)	0.26
Psychotic disorder (ref. No)	0.89 (0.27-2.91)	0.85
Alcohol use disorder (ref. No)	1.12 (0.57-2.21)	0.75
Substance use disorder (ref. No)	1.31 (0.66-2.59)	0.44
Alcohol use disorder or Substance use disorder (ref. No)	0.98 (0.49-1.95)	0.94
Anxiety disorders (ref. No)	1.82 (0.91-3.62)	0.09
Post-traumatic stress disorder diagnosis (ref. No)	3.51 (1.76-7.01)	<0.001
Substance Use		
Substance use severity score, past year	1.00 (0.99-1.01)	0.85
Tobacco use, current (ref. No)	0.34 (0.15-0.77)	0.01
Clinical Characteristics		
Anxiety	1.04 (0.98-1.11)	0.20
Depression	1.05 (0.99-1.11)	0.10
Hopelessness	0.97 (0.83-1.12)	0.65
Physical abuse	1.04 (0.98-1.11)	0.20
Sexual abuse	1.00 (0.94-1.05)	0.92
Childhood trauma questionnaire total	1.00 (0.97-1.03)	0.96
Post-traumatic stress disorder symptoms	1.02 (1.00-1.04)	0.07
Aggression	0.69 (0.07-6.59)	0.75
Affective lability	1.11 (0.67-1.83)	0.68
Apathy	1.08 (1.03-1.13)	0.002
Reasons for living	0.99 (0.98-1.00)	0.01
Suicide ideation	1.01 (1.00-1.02)	0.01
Impulsivity	1.02 (0.99-1.05)	0.22

© 2023 Thoma BC et al. *JAMA Network Open.*

Social support	0.74 (0.57-0.96)	0.02
Perceived stress	1.02 (0.95-1.08)	0.61
Anhedonia	1.13 (1.01-1.27)	0.04
Symptomatology	1.01 (1.00-1.02)	0.12
Lifetime History		
Suicidal behavior (ref. No)	5.51 (0.75-40.34)	0.09

[a]HR = hazard ratio; CI = confidence interval

© 2023 Thoma BC et al. *JAMA Network Open.*
