# Supplementary material for: Risk for Suicidal Behavior After Psychiatric Hospitalization Among Sexual and Gender Minority Patients
Source: JAMA Netw Open. 2023 Sep 8;6(9):e2333060. doi: 10.1001/jamanetworkopen.2023.33060 (PMC10492186; doi:10.1001/jamanetworkopen.2023.33060)
Supplement: Supplement 2. — Data Sharing Statement [file jamanetwopen-e2333060-s002.pdf]

Data Sharing Statement

Thoma. Risk for Suicidal Behavior After Psychiatric Hospitalization Among Sexual and Gender Minority Patients. *JAMA Netw Open*. Published September 08, 2023. doi:10.1001/jamanetworkopen.2023.33060

Data
Data available: No

Additional Information
Explanation for why data not available: Due to risk of deductive disclosure with stigmatized, minoritized populations, data are only available via a data sharing agreement with the investigative team.
